# Factors Related to Cardiovascular Disease Risk Reduction in Midlife and Older Women: A Qualitative Study

**Published:** 2007-12-15

**Authors:** Sara C Folta, Jeanne P Goldberg, Rebecca Seguin, Peter N Reed, Miriam E Nelson, Alice H Lichtenstein

**Affiliations:** John Hancock Center for Physical Activity and Nutrition, Friedman School of Nutrition Science and Policy, Tufts University; John Hancock Center for Physical Activity and Nutrition, Friedman School of Nutrition Science and Policy, Tufts University, Boston, Massachusetts; John Hancock Center for Physical Activity and Nutrition, Friedman School of Nutrition Science and Policy, Tufts University, Boston, Massachusetts; John Hancock Center for Physical Activity and Nutrition, Friedman School of Nutrition Science and Policy, Tufts University, Boston, Massachusetts; John Hancock Center for Physical Activity and Nutrition, Friedman School of Nutrition Science and Policy, Tufts University, Boston, Massachusetts; the Friedman School of Nutrition Science and Policy and the Jean Mayer USDA Human Nutrition Research Center on Aging, Tufts University, Boston, Massachusetts.

## Abstract

**Introduction:**

Cardiovascular disease (CVD) is the leading cause of death for women in the United States. A healthy diet and appropriate physical activity can help reduce the risk for CVD. However, many women do not follow recommendations for these behaviors. In this study, we used qualitative methods to better understand knowledge and awareness about CVD in women, perceived threat of CVD, barriers to heart-healthy eating and physical activity, and intervention strategies for behavior change.

**Methods:**

We conducted four focus groups with 38 white women aged 40 years or older in Kansas and Arkansas. We also interviewed 25 Cooperative State Research, Education, and Extension Service agents in those states. Environmental audits of grocery stores and the physical environment were done in three communities.

**Results:**

Most women were aware of the modifiable risk factors for CVD. Although they realized they were susceptible, they thought CVD was something they could overcome. Common barriers to achieving a heart-healthy diet included time and concern about wasting food. Most women had positive attitudes toward physical activity and reported exercising in the past, but found it difficult to resume when their routine was disrupted. The environmental audits suggested that there are opportunities to be physically active and that with the exception of fresh fish in Kansas, healthful foods are readily available in local food stores.

**Conclusion:**

Interventions to change behavior should be hands-on, have a goal-setting component, and include opportunities for social interaction. It is especially important to offer interventions as awareness increases and women seek opportunities to build skills to change behavior.

## Introduction

Heart disease is still considered a disease that affects men, although every year since 1984, it has affected more women than men in the United States (1). In 2004, approximately 500,000 women died of cardiovascular disease (CVD), making it the leading cause of death for women in this country (2).

A lifestyle that includes a healthy diet, weight control, and appropriate physical activity can dramatically reduce the risk of heart disease in women (3-9). A dietary pattern that focuses on vegetables, fruits, low and nonfat dairy foods, whole grains, legumes, fish, and lean meats helps to reduce cholesterol levels and lower blood pressure, leading to an overall reduction in CVD risk (1,10-12). Increasing physical activity similarly helps to improve weight control and reduce risk of developing CVD in women (13). Yet few women are leading heart-healthy lifestyles. According to the 1999–2000 National Health and Nutrition Examination Survey data, half of women aged 51–70 years fail to eat at least 5 servings of fruits and vegetables per day (14). Nearly 40% of women do not engage in any type of leisure-time physical activity (15), and nearly 70% of women aged 40 or older are overweight or obese (16).

Strategic tactics to reduce CVD risk involve the development and evaluation of educational and behavioral programs that can be implemented by organizations in communities where many women at high risk can be reached. To develop effective interventions, it is important to understand the target population in relation to the behaviors. Qualitative methods are ideal for gathering in-depth information to help develop this understanding (17). By using several different qualitative methods, the findings of each may be confirmed and extended (18).

Several previous studies have used qualitative methods to examine women's perceptions and awareness of CVD risk. Focus groups have been conducted with low-income African American women (19-22) as well as young and middle-aged white, Latina, and American Indian women (21-23). The results of these studies suggest that awareness of personal risk varies in different populations. Common barriers to behavioral change to reduce risk include a lack of support, food preferences, time, and cultural factors. Women in these studies said they wanted interventions that taught them skills, were tailored to their needs and situations, and included social support.

CVD develops over several decades, and efforts to prevent it that begin early in life are likely to have the greatest benefit. However, lifestyle modifications may still reduce risk, even in older adults (24). These efforts may become especially important as the United States faces a growing number of older citizens (25).

In this study, focus groups were conducted with midlife and older (aged 40 or older) sedentary women who would be appropriate candidates to target with an intervention. The objectives of this study were to use qualitative methods to determine the knowledge and awareness of CVD risk in midlife and older women, identify barriers to heart-healthy eating and physical activity, and develop intervention strategies that are likely to be feasible and effective.

## Methods

### Focus groups

We conducted four focus groups in Kansas and Arkansas in June 2006. Two groups were conducted in each state: one in a rural community (population of less than 7000) and one in a small city (population of approximately 40,000). Cooperative State Research, Education, and Extension Service (CSREES) agents, who serve as leaders on health issues in rural communities, recruited a purposive, nonrandom sample of sedentary women aged 40 or older. Women were recruited through CSREES agents' community networks and through listings at community events. Focus groups took place at CSREES sites within the communities and were led by a trained focus group facilitator. In total, 38 women participated, with group sizes ranging from 8 to 11 participants. Sessions typically lasted 90 minutes. A $50 incentive was given to each participant to improve attendance. Each session was recorded on a digital audio recorder for subsequent transcription. Participants signed informed consent forms in accordance with the requirements of the Tufts University Institutional Review Board.

The discussion guide for the focus groups was designed to address four key topic areas: 1) awareness and knowledge about CVD risk factors; 2) attitudes, perceptions, and barriers regarding physical activity; 3) attitudes, perceptions, and barriers regarding a heart-healthy diet; and 4) opinions about nutrition and physical activity interventions. We conducted a pilot focus group using the guide to ensure good discussion flow and question comprehension. No changes were made to the guide after the pilot.

The NVivo program (version 2.0 for Windows, QSR International Pty Ltd, Doncaster, Victoria, Australia) was used to help code the data. One person coded key phrases into a framework that was based on the questioning structure. During this initial coding process, additional themes emerged from the data and were added to the framework. Data were then recoded using the revised framework.

### Interviews with CSREES agents

CSREES, part of the U.S. Department of Agriculture, has as its mission the advancement of knowledge for agriculture, the environment, and human health and well-being (26). The food, nutrition, and health programs within CSREES are designed to strengthen the nation's capacity to address issues related to diet, health, food safety, food security, and food science and technology (27). Because of their role, CSREES agents have in-depth knowledge of the communities they serve and are in an ideal position to deliver interventions related to heart health.

Two members of the research team conducted structured interviews with 15 CSREES agents in Arkansas and with 10 CSREES agents in Kansas. The discussion guide for these interviews was designed to determine their perceptions of the target population regarding nutrition, physical activity, and heart health, and to obtain their opinions on interventions to address these issues. All of the agents were women. They represented a wide range of geographic locations within each state. However, in accordance with the CSREES mission, most were in rural communities.

Interviews with each agent were conducted by telephone and lasted 15 to 30 minutes. The responses were compiled in a word processing program, and the NVivo program was then used to assist with coding. As with the focus group data, the data from these interviews were coded in a two-step process: key phrases were coded into a framework that was based on the questioning structure, and additional themes that emerged from the data were added to the framework and coded. CSREES agents signed informed consent statements approved by the Tufts University Institutional Review Board.

### Community observation

The research project manager used community observation to confirm and extend the information gathered through the focus groups and interviews and to assess the availability of specific food items. Three of the four communities represented in the focus groups, the two in Kansas and the larger one in Arkansas, were observed. An unforeseen transportation issue prevented observation of the fourth community. Observation included an audit of the major supermarkets in the community as well as health food stores, if there were any. We identified stores through online business directories and by asking the county CSREES agents, all of whom had resided in the communities for many years. To guide the audit, we developed a list of foods that might be considered "heart healthy." It included whole grain pasta, bread products and flours, and brown rice; a variety of fresh and frozen produce; dried and canned beans; canned, fresh, and non-breaded frozen fish (any type); and low-fat dairy (how much 1% or nonfat milk was available in proportion to 2% or whole milk). The research project manager visited all stores and checked whether the items on the list were available in the store. In addition, digital photographs were taken to document how these foods were presented in the stores.

The research project manager also observed the physical environment. This part of the audit was based on the Irvine Minnesota Inventory for Observation of Physical Environment Features Linked to Physical Activity (28). The project manager used the coding instrument from this inventory as a guide to determine accessibility (e.g., easy to get to, no locked gates or other barriers), pleasurability, perceived safety from traffic, and perceived safety from crime in the main downtown area and in major residential sections of the communities. The information gathered included the availability of a public recreation area, the condition of sidewalks, and the presence of crosswalks, curb cuts, and pedestrian crossing signals. However, it was not a formal, quantitative audit, because a single data collector made observations, and systematic sampling was not used. The goal was to use a set of standard questions to form qualitative impressions of the physical environment.

## Results

The recruitment criteria for the focus groups were sex, age, and physical activity level. Women were required to be at least 40 years old and sedentary. The participants who met those criteria and responded ranged in age from early 40s to late 80s. Reflecting the demographics of the communities, all women were white.

### Knowledge and awareness about CVD risk in women

CSREES agents described the women in the target population as having a variable level of awareness about heart disease risk in women, and focus group data supported this. Most women in the focus groups were aware that the leading cause of death for women in the United States is heart disease, although several believed that it was breast cancer. They were generally aware that heart attack symptoms for a woman are often more subtle than those for men. One group talked about women having smaller veins. They were aware of both modifiable risk factors and the genetic component for CVD.

Participants in all focus groups identified a number of foods as being part of a heart-healthy diet, including low-fat foods. Whole grains (oatmeal in particular) and fruits and vegetables were named in all four groups. Nuts, beans, and fish that is not fried were also mentioned.

CSREES agents reported that the women were more likely to have misconceptions about diet than about physical activity. The agents' perceptions were that misconceptions were likely to be about the role of *trans* fats and about fad dieting. However, focus group participants were aware that *trans* fats should be avoided, and no misconceptions about them emerged. Participants did have some food-related misconceptions, though. Cheese, garlic, and spices were incorrectly named as foods that would promote heart health. Coffee and caffeine were incorrectly named as things that should be avoided.

The women talked about several types of physical activity that would be good for their hearts, including walking, running, or things that "get your heart rate up." CSREES agents confirmed that for the most part, women in the target population have a moderately high level of understanding about the role of physical activity in reducing risk and the types of activity that are most beneficial, but they have difficulty in putting their knowledge into practice.

### Perceived threat of CVD

Many women in the four groups said that their greatest concern about their own health was not a specific illness, but developing any condition that would incapacitate them.

I think it would be horrible to be incapacitated where you couldn't do for yourself . . . you couldn't drive, you couldn't walk to the mailbox, or whatever, you had to depend on someone else to do it for you. (larger community, Arkansas)

Many saw CVD as something that could be overcome, and they were not concerned about it despite their awareness that heart disease is the leading cause of death among women.

We have a lot of heart history in our family, too, but they've survived it. And they've had stents and bypasses and all of this, but they've survived it and are doing very well — cancer just seems to be one of those things that you can't get stopped . . *.* (larger community, Kansas)

I've been there, and done that, been through two major heart surgeries, and I'm invincible. (larger community, Arkansas)

CSREES agents confirmed that heart disease was not perceived as a major threat to women, despite their high levels of awareness. Agents added that some women are more concerned about breast cancer and that other women believe that heart disease will not happen to them.

Some focus group participants expressed a certain amount of fatalism regarding their risk, because of strong family history. In each group, at least one woman talked about how diet and physical activity had not made an impact on her cholesterol levels. These women still thought it was a good idea to eat healthfully, exercise, and get checked by a doctor so that they would not have to worry about it as much.

My goal for myself is just to make changes that are healthy and become so much a part of my life that I'm not focused on that. (Several agree). I'd rather be focused on a lot of other things. (smaller community, Kansas)

### Barriers to healthy eating

Only one community (in Kansas) had a health food store. However, the results from the audit suggest that most heart-healthy foods are readily available in the communities and that access is not a major barrier. The major supermarkets had a good selection of whole grain products. They also had a good selection of fresh and frozen vegetables, fresh and frozen fruits, and dried and canned beans. Although the stores devoted more space to 2% and whole milk, all had an ample supply of 1% and nonfat milk. All stores had a good selection of canned fish. Fresh fish was readily available in Arkansas, but in Kansas, only the one large store in the larger city had fresh fish. Stores in both states carried frozen fish, but breaded fish dominated the freezer section, and the selection of plain filets was extremely limited.

Data from the CSREES agent interviews and focus groups corroborated the results of the environmental audit, although a few women added that fresh produce is not as readily available in the winter months. Although heart-healthy foods are readily available, women said that they find it difficult to avoid less healthful foods.

And the healthy foods are always there. You know, you can lead a horse to water but can't make him drink. I try to cook healthy and try to have healthy things . . . but I like fried foods too, so it's hard. (larger community, Kansas)

Many women reported that avoiding high-calorie snacks was especially difficult. They saw snacking as their main downfall. Even when they were able to eat more healthfully at meals, they reported having difficulty choosing healthy snacks.

Time emerged as a major barrier to healthy eating, for different reasons. Women with children still in the home said that they had very busy schedules and did not have time to cook. Retired women said that they were tired of cooking after doing it for so many years and did not want to spend the time.

Women who lived with husbands and children thought that it would be easier for single women to eat a more healthful diet.

I think when you have kids, there's a snack problem. We still have a child at home, and he will eat salads and vegetables, but he really likes to have other things in the house, too. (smaller community, Kansas)

Conversely, women who lived alone thought it would be easier for those with husbands and families to eat better.

I think I'm one of the oldest ones here, so I can say as a younger mother, I did that [cooked healthfully] for my family. Trying to have them have a healthy diet. But now, it's a lot harder. (larger community, Kansas)

Wasting food came up as a barrier to change in three of the four groups. Women reported eating more than they want because they do not want to throw food away.

And we're in a generation, our kids now are not that way, but we're in a generation that don't waste food. I mean, my kids were — when they went to the table and they ate what was on the table and they cleaned their plates out. But now, they're not that way. So I think that's an example, because we've been taught not to waste food and we eat instead of throwing it out. (larger community, Arkansas)

Other barriers included being pressured to eat at social events, confusion over what they perceive as conflicting health messages, hunger when they try to cut down on portion sizes, lack of menu planning that leads to eating out, not liking fruits and vegetables, and difficulty in changing eating patterns they had developed in childhood.

### Barriers to physical activity

In the communities in both states, the overall qualitative impression from the environmental audit was that there were readily accessible, pleasant places to walk that were reasonably safe in terms of both traffic and crime. In Arkansas, there were very few sidewalks in rural areas, but it was still possible to walk safely. CSREES agents confirmed these observations.

Weather did arise as a barrier to physical activity in both the focus groups and the key informant interviews. In terms of indoor physical activity, the three communities that were observed all had gyms. Some focus group participants said that feeling self-conscious at the gym was also a barrier. There were other options for indoor walking in all communities.

Most CSREES agents felt that most women would be willing to increase their physical activity levels. Many of the focus group participants had engaged in regular physical activity in the past but found it difficult to resume after something disrupted their routine.

[A]nd then I changed jobs, and it took so long to get down to the Y to work out . . . I just stopped doing it, and then gradually I just started eating bad again and whatnot . . . I don't really have an excuse now. I have lots of time, I could do it, I just got out of the habit. (larger community, Arkansas)

A few women said that physical activity could be boring, but they would be willing to do it if it could be made fun. Only a couple of women said that they do not exercise because they are lazy or dislike it. Even those women seemed somewhat willing to try it if they could find something that they would enjoy. A few women did not want yet another commitment, and they mentioned strategies for incorporating physical activity into their regular schedule, such as parking further away from the store or taking the stairs rather than the elevator.

### Intervention strategies

Overall, the women felt that they already knew a fair amount about what they need to do to reduce their risk of CVD in terms of diet and physical activity but that they just need help putting that knowledge into practice. They said that to motivate them to keep coming, an intervention program should be hands-on. CSREES agents confirmed that programs with a hands-on component are most popular with their constituency. Hands-on nutrition intervention programs that had worked best for them in the past had included tastings and cooking exercises.

Focus group participants also wanted a program to include goal-setting where they set reasonable, realistic goals so that they could see results, even small ones. They wanted to receive recognition that they had met those goals. CSREES agents felt comfortable in helping women set goals and in giving them recognition for meeting goals.

CSREES agents and focus group participants both reported that walking is a preferred form of physical activity. Most women expressed a positive attitude about both walking and dancing, especially when they could be done with other people. CSREES agents confirmed that their most successful programs for midlife or older women include a social component, and that if participants have opportunities to build relationships, they will be highly motivated to keep coming back.

## Discussion

There was a high level of awareness of CVD among the women in the focus groups in this study. Two large national campaigns, the National Heart, Lung, and Blood Institute's Heart Truth campaign (29) and the American Heart Association's Go Red for Women campaign (30), have put substantial resources toward increasing awareness in recent years. Awareness has increased significantly since the Go Red for Women campaign began in 1997 (31). Although women were not specifically asked how they had heard about the problem, these campaigns could have contributed, either directly or indirectly, to the level of awareness in this population.

Both the focus groups and the interviews with CSREES agents indicate that the women are knowledgeable about CVD risk factors. Although there were some misconceptions, especially concerning diet, they were few. The women's belief that low-fat foods are inherently protective against CVD probably reflects older messages about CVD, which focused on total fat rather than saturated fat. It is uncertain why cheese was mentioned as a heart-healthy food. Several women believed that coffee or caffeine should be avoided. Although a study done in 1957 demonstrated a relationship between drinking coffee and CVD and was influential for many years, the current evidence is mixed and inconclusive (32).

Although the women recognized that they had a high level of susceptibility to CVD, they perceived the severity of CVD to be low. They saw CVD as something that they could either live with or overcome. At the same time, their biggest concern regarding their health was about becoming disabled in some way. This suggests that health messages that focus on CVD as a potentially disabling condition may help persuade women to take action by increasing their perception of the potential severity of CVD.

Waste emerged as a major barrier to dietary behavior change. Women said that they would eat more than they wanted because they object strongly to letting food go to waste. This did not come up as an issue in previous studies with younger women and could reflect the age demographic in the groups. This belief should be taken into account when designing interventions targeted at older people.

In other qualitative studies, family responsibilities and preferences emerged as major barriers to physical activity and heart-healthy eating (22,23). These themes were not prominent in our results, perhaps because the women were older and either lived alone or had older children with less influence on their time and on the family meal. However, the time necessary to purchase and prepare food did emerge as a barrier. In our study, retired women reported having the time, but not wanting to take it. Food preference, a barrier for women in previous studies, also surfaced in our groups. This is not surprising, because taste is a major determinant of food choice (33).

Overall, the results suggest that many of the women were in the contemplation stage according to Prochaska's Transtheoretical Model (34). According to the model, people in this stage intend to change in the next 6 months. They are aware of the benefits of change but are also acutely aware of the costs and are in the process of balancing the two. Most women in the focus groups were aware of the problem of CVD and knowledgeable about diet and exercise, suggesting that they had actively sought out information. Many had exercised in the past and had had positive experiences, but had fallen out of the habit. CSREES agents described them as being willing to increase their physical activity levels. However, both CSREES agents and the women themselves spoke of the many barriers to making a change. The women had difficulty putting their knowledge into action. This finding may reflect the way participants were recruited. Although it was specified that they must be sedentary to participate, women with an interest in diet, physical activity, and CVD who were willing to discuss these issues without taking part in an action-oriented program were probably more likely to respond.

Self-reevaluation strategies may be appropriate and effective for women in this stage (34). These have been effective in moving people from the contemplation stage to the preparation stage, in which a person intends to take action in the next month and has taken some significant action in the past year (34). Self-reevaluation techniques may help a woman see how the benefits outweigh the costs by causing her to evaluate her self-image when she is doing the changed behavior ("I feel like a strong person when I exercise") or when she is not ("I feel lazy and unhappy when I don't exercise"). Self-reevaluation techniques include the provision of healthy role models, imagery, and value clarification (35).

Both the focus group and the interview results suggest that a viable intervention should include hands-on strategies such as taste testing and food preparation, allow space for social interaction, and include a goal-setting component. The community observations indicate that the environment will support positive behavior change. Foods that fit into a heart-healthy eating pattern are readily available, with the exception of fresh fish in Kansas. Walking is a preferred form of physical activity, and there are safe and pleasant places to walk. Dancing may be a good alternative when exercise must be done indoors.

These results contribute to a growing body of evidence about women's knowledge and perceptions regarding CVD risk. They also provide some guidance for preferred strategies for behavior change. This is especially important as awareness increases and women look for opportunities to develop the skills necessary to help reduce their risk of this serious disease.

## Acknowledgments

This work was supported by a research grant from the Fannie E. Rippel Foundation.
